# NF-κB1 p50 stabilizes HIF-1α protein through suppression of ATG7-dependent autophagy

**DOI:** 10.1038/s41419-022-05521-1

**Published:** 2022-12-27

**Authors:** Junlan Zhu, Shirui Huang, Yang Li, Jiheng Xu, Ruifan Chen, Mengxin Guo, Xiaohui Qian, Tengda Li, Zhongxian Tian, Honglei Jin, Chuanshu Huang

**Affiliations:** 1 Oujiang Laboratory (Zhejiang Lab for Regenerative Medicine, Vision and Brain Health), Key Laboratory of Laboratory Medicine, Ministry of Education, School of Laboratory Medicine and Life Sciences, Wenzhou Medical University, 325035 Wenzhou, Zhejiang China; 2 Precision Medicine Laboratory, Beilun People’s Hospital, Beilun Branch of the First Affiliated Hospital, School of Medicine, Zhejiang University, 315800 Ningbo, Zhejiang China

**Keywords:** Macroautophagy, Transcriptional regulatory elements

## Abstract

The function and underlying mechanisms of p50 in the regulation of protein expression is much less studied because of its lacking of transactivation domain. In this study, we discovered a novel function of p50 in its stabilization of hypoxia-inducible factor 1α (HIF-1α) protein under the condition of cells exposed to arsenic exposure. In p50-deficient (p50^−/−^) cells, the HIF-1α protein expression was impaired upon arsenic exposure, and such defect could be rescued by reconstitutional expression of p50. Mechanistic study revealed that the inhibition of autophagy-related gene 7 (ATG7)-dependent autophagy was in charge of p50-mediated HIF-1α protein stabilization following arsenic exposure. Moreover, p50 deletion promoted nucleolin (NCL) protein translation to enhance *ATG7* mRNA transcription via directly binding transcription factor *Sp1* mRNA and increase its stability. We further discovered that p50-mediated miR-494 upregulation gave rise to the inhibition of p50-mediated NCL translation by interacting with its 3’-UTR. These novel findings provide a great insight into the understanding of biomedical significance of p50 protein in arsenite-associated disease development and therapy.

## Introduction

As the valuable tumor therapeutic agents, arsenic trioxide could induce apoptosis in several cancer cells [1], however, occupational or environmental exposure to arsenic remains a major public health problem. As a carcinogen, chronic arsenic exposure is associated with a few human cancer development, such as skin, bladder, liver, and lung cancers [2]. It has been thought that arsenic-induced the generation of reactive oxygen species (ROS), leading to the generation of oxidative stress and activation of various signaling cascade and gene expression, which is the key event eventual resulting in carcinogenic effects on human [3].

Hypoxia-inducible factor 1 (HIF-1) is regarded as the important transcriptional regulator which controls expression of several genes, including genes involved in oxygen transport, angiogenesis, and glucose metabolism, therefore HIF-1 is critical in cellular adaptations to hypoxia [4]. The regulatory subunit HIF-1α and the constitutively expressed subunit HIF-1β are composed together to be the HIF-1 heterodimer, which is an active transcription factor responsible for its regulated gene expression. A prerequisite for the HIF-1 heterodimer formation is the hypoxia-dependent stabilization of the HIF-1α subunit and its co-activators recruitment. This is mainly achieved by hydroxylation at the post-translational level, which occurred at the HIF-1α N-terminal and C-terminal transactivation domain [5, 6]. Furthermore, mechanisms underlying to phosphorylation and promoting HIF-1α itself gene transcription also contribute to enhancing its protein expression level [7].

As a transcription factor protein complex, NF-κB mediates a series of genes transcription regulation [8]. In mammalian cells, there are five subunits in the NF-κB family, including p50 (NF-κB1), p52 (NF-κB2), p65 (RelA), RelB, and c-Rel [9]. In compared to other members of NF-κB family, p50 is much less studied because of its lacking of transactivation domain. The studies from our group revealed that p50 itself alone could induce GADD45α protein and promote p53 protein translation upon arsenic exposure [10, 11]. In this study, we discovered that p50 stabilized HIF-1α protein by suppressing ATG7-dependent autophagy.

Autophagy is a strictly and evolutionarily conserved process that degrades cytoplasmic organelles and material [12]. Oxidative stress, starvation, or other harmful conditions could induce and upregulate autophagy function [13]. Autophagy-related genes (ATG) participate in the formation of autophagosomes [14]. As an E1-like activating enzyme, ATG7 activates the ATG8 family of ubiquitin-like proteins (UBLs) and the ATG12 UBL, facilitating their conjugation to the lipid phosphatidylethanolamine and ATG5, respectively, and forms the two ubiquitin-like systems [15–17]. Here, we discovered a crucial role of p50 in NF-κB transcription-independent cascade to attenuate the arsenite-treated cells to autophagy, by which attenuated the HIF-1α protein degradation.

## Materials and methods

### Reagents and plasmids

Arsenite (As^3+^) was bought from Aldrich (Milwaukee, WI, USA). Actinomycin D (Act D), proteasome chemical inhibitor MG132, and protein synthesis inhibitor cyclohexamide (CHX) were purchased from Calbiochem (San Diego, CA, USA), Bafilomycin A1 was purchased from Santa Cruz (St. Louis, MO, USA). TRIzol reagent, the dual-luciferase assay kit, and SuperScript™ First-Strand Synthesis system were bought from Promega (Madison, WI, USA) and Invitrogen (Grand Island, NY, USA), respectively. Poly Jet™ DNA In Vitro Transfection Reagent was obtained from SignaGen Laboratories (Rockville, MD, USA). The plasmid of human ATG7 promoter (from −1398 to −227)-driven luciferase reporter was constructed with Kpn I and Bgl II using genomic DNA based on NCBI database. Human ATG7 promoter mutant luciferase reporter (the binding site of Sp1 was mutated) was cloned into the pGL3 basic luciferase reporter vector. The GFP-LC3B and its control vector were a kind gift from Dr. Gang Chen (University of Kentucky, Lexington, KY, USA) [18]. The shRNA plasmids specifically targeting p50, NCL, Sp1, Beclin1, and ATG7 were bought from Open Biosystems Inc. Construct expressing miR-494 was a gift from K. Yoshida (Department of Life Sciences, Meiji University, Kanagawa, Japan) [19]. GFP-NCL expression vector was a generous gift from Dr. Michael B. Kastan (Duke University School of Medicine, Duke Cancer Institute, Durham, NC) [20]. Human *NCL* 3’-UTR luciferase reporter was cloned into the pMIR luciferase assay vector. All plasmids were prepared by the Plasmid Preparation/Extraction Maxi kit from QIAGEN (Valencia, CA, USA).

### Cell culture and transfection

Beas-2B cell lines were originally obtained from ATCC; both cell lines are regularly authenticated every 6–12 months on the basis of viability, recovery, growth, morphology, and chemical response as well as by testing STR loci and gender using the PowerPlex® 16 HS System. The final results will be compared with data from the ATCC STR DATABASE. The cells were cultured at 37 °C with 5% CO_2_ in Dulbecco’s modified Eagle’s medium (DMEM; 11995065) supplemented with 10% fetal bovine serum (FBS; 26140079), 1% penicillin/streptomycin (15140163), 2 mM L-glutamine (25030164), all were purchased from Life Technologies [21]. The p50^−/−^ and p65^−/−^ MEFs and their corresponding wild-type (WT) MEFs were cultured as described in our previous reports [10]. The stable cell lines of p50^−/−^(p50) and p65^−/−^(p65) were established and described in our previous publications [10]. Mouse epidermal JB6 Cl41 cells were cultured in MEM with 5% FBS. Cell transfections were performed by using PolyJet DNA In Vitro Transfection Reagent, according to the manufacturer’s instruction. For stable transfection, cultures were subjected to either puromycin (Alexis, Plymouth, PA, USA) or G418 (Invitrogen, Carlsbad, CA, USA) selection. The surviving cells that from the drug selection were pooled as stable mass culture.

### Antibodies and western blot analysis

Antibodies against HIF-1α were purchased from Novus Biologicals, Inc. (Littleton, CO) (APR07745G); The antibodies against GADD45α (sc-6850), p65 (sc-8008), p50 (sc-8414), NCL (sc-55486), Sp1 (sc-420) were obtained from Santa Cruz Biotechnology Inc. Anti-COX-2 antibody (No. 160107) was purchased from Cayman Chemical. The antibodies specific against ATG3, ATG5-ATG12, ATG7, Beclin1, LC3A, LC3B (Autophagy Antibody Sampler Kit #4445), ATG16L1 (#8089), P62 (#8025), ELAVL1 (#12582), Elk1 (#9182), Ets1 (#14069), STAT5 (#25656), and GAPDH (#5174) were bought from Cell Signaling Technology (Beverly, MA, USA). The antibody specific against HNRNPD (OAAF01114) was purchased from Aviva Systems Biology (San Diego, USA), while antibodies against β-Actin (A5316) were purchased from Sigma (St. Louis, MO, USA). Western blot was performed as described in our previous publication [22]. The relative band intensity was analyzed using the Image Lab software (Bio-Rad).

### Quantitative RT-PCR for mRNA and miRNA assay

The indicated cells were treated with arsenite for 24 h, and the cells were then used for total miRNA extraction using the miRNeasy Mini Kit (Qiagen, Valencia, CA, USA). Total RNA (2.0 μg) was used for reverse transcription. The analysis of miRNA expression was carried out using the miScript PCR system (Qiagen, Valencia, CA, USA) and the 7900HT Fast Real-Time PCR system (Applied Biosystems, Carlsbad, CA, USA). The initial activation was performed at 95 °C for 15 min, followed by 40 cycles of denaturation at 95 °C for 15 s, annealing at 55 °C for 30 s, and extension at 72 °C for 30 s. Cells were cultured as described same as for miRNA extraction, 5.0 μg total RNA was used for first-strand cDNA synthesis with oligod T (20) primer by SuperScriptTM First-Strand Synthesis system (Invitrogen, 11904018). The analysis of mRNA expression was performed by using the Fast SYBR Green Master Mix kit (Applied Biosystems, 4385614) in the 7900HT Fast Real-Time PCR System (Applied Biosystems, Foster City, CA, USA). The primers used in this study were: human *HIF-1α* (Forward, 5’-TGG TCA AAT CGG CCT CAG CA-3’; Reverse, 5’-CCC TGA ACG GAG GCA TTG GC-3’) and human *β-Actin* (Forward, 5’-CTC CAT CCT GGC CTC GCT GT-3’; Reverse, 5’-GCT GTC ACC TTC ACC GTT CC-3’), human *ATG7* (Forward, 5’-ACC CGG CTC ACC CTG GTT TGT-3’; Reverse, 5’-ACC CCA GTC CTG TAG GTG TGC TG-3’), human *Sp1* (Forward, 5’-AAA TTG AAT GGG AAG GTG AT-3’; Reverse, 5’-GAA CCC ACG CCT CTT ATT G-3’), human *NCL* (Forward, 5’-ACC CGG CTC ACC CTG GTT TGT-3’; Reverse, 5’-ACC CCA GTC CTG TAG GTG TGC TG-3’). Data were analyzed as described in the previous publication [23].

### Luciferase reporter assay

ATG7 promoter-driven luciferase, ATG7-mutated promoter-driven luciferase (Sp1 binding site was mutated) or *NCL* 3’-UTR luciferase reporter plasmids were transiently transfected into cells. The transfectants were seeded into each well of 96-well plates (8 × 10^3^ cells per well) and subjected to the various treatments, as described in our previous study [24]. Luciferase activities were determined with the Dual-Luciferase Reporter Assay System using a luminometer as described previously [25].

### Fluorescence microscopy

p50^−/−^ and its WT cells were cultured on cover slides in 10% FBS DMEM medium for 48 h. The cells were exposed to 20 μM arsenite for the indicated time and fixed with 4% paraformaldehyde (Sigma–Aldrich Corporation, 158127) in PBS (135 mM NaCl, 4.7 mM KCl, 10 mM Na2HPO4, 2.0 mM NaH2PO4, pH 7.4) at room temperature for 15 min, and then stained with 0.1 mg/ml DAPI (Sigma–Aldrich Corporation, 9542) for 1 min. The slides were washed three times with PBS and mounted with anti-fade reagent (Molecular Probes, P36930). All the cell images were captured using an inverted Leica fluorescence microscope (Wetzlar, Germany). For quantification of autophagic cells, GFP-LC3B puncta were determined by counting at least 30 cells per slide [26, 27].

### RNA-IP assay

293 T cells were cultured in 10 cm dishes. When cell confluence reached 70–80%, cells were transiently transfected with GFP-NCL and its vector control. Twenty-four hours after the transfection, the cells were extracted with polysomelysis buffer (10 mM HEPES pH 7; 100 mM KCl; 5 mM MgCl_2_; 25 mM EDTA; 0.5% IGEPAL; 2 mM DTT; 50 units/ml RNase OUT; 50 units/ml Superase IN; 0.2 mg/ml heparin; and complete proteinase inhibitor). The cell lysates were centrifuged at 14,000 × *g* for 10 min at 4 °C. The anti-GFP agarose beads A/G (Vector laboratories, Burlingame, CA, USA) were added into the supernatant and rotated overnight at 4 °C in NET2 buffer (50 mM Tris-HCl, pH 7.4, 150 mM sodium chloride, 1 mM magnesium chloride, 0.05% IGEPAL, 50 U/mL RNase OUT, 50 U/mL Superase IN, 1 mM dithiothreitol, and 30 mM EDTA). The beads were washed three times, and resuspended in 100 μL NET2 and 100 μL SDS-TE (20 mM Tris-HCl, pH 7.5, 2 mM EDTA, and 2% sodium dodecyl sulfate) and then incubated at 55 °C for 30 min, mixing occasionally. The RNAs in the buffer of the beads were extracted by phenol-chloroform- isoamyl alcohol and RT-PCR was performed to identify the mRNA presented in the immune-complex.

### The construct of human ATG7 promoter-driven mutant luciferase reporter and NCL mRNA 3’-UTR luciferase reporter

The plasmid of ATG7 promoter (from −1398 to −227)-driven luciferase reporter was constructed by amplifying from genomic DNA isolated from Beas-2B cells based on NCBI database (NC_000003.12), using primers: forward, 5’-ACT GGT ACC ACT GAC ACA CAC AAC CCC TAC TGA G-3’, and reverse, 5’-ACT AGA TCT GAG AGG CGG CAT CAA ACG CAG CAC A-3’. A three-point mutation was introduced into the seed region of Sp1/ATG7 promoter putative interacting sequence using primers: MIRMUTFOR, 5’-TCC TGA CCT CGT GAT CCA TAC GCC TCG GCC TCC CAA-3’ and MIRMUTREV, 5’-TTG GGA GGC CGA GGC GTA TGG ATC ACG AGG TCA GGA-3’, according to the site directed mutagenesis protocol (Quick-Change Site Directed Mutagenesis, Stratagene), for producing the pGL3-ATG7 promoter mutant plasmid. The plasmid of *NCL* 3’-UTR luciferase reporter was constructed by amplifying from cDNA isolated from Beas-2B cells based on NCBI database, using primers: forward, 5’-ACT GGT ACC ACT GAC ACA CAC AAC CCC CTA CTG AG-3’ and Reverse, 5’-ACT AGA TCT GAG AGG CGG CAT CAA ACG CAG CAC A-3’), and then subcloned into the Kpn I and Bgl II sites into pMIR vector, thus originating the pMIR-*NCL* 3’-UTR luciferase reporter plasmid. Constructs were all sequencing verified by GENEWIZ (South Plainfield, NJ).

### Statistical analysis

Statistical analyses were performed using the Prism 8.0.2 software (GraphPad Software, USA). Quantitative data were compared using a Student’s *t*-test between two samples or one-way analysis of variance (ANOVA) for multiple samples. All results were indicated as the mean ± S.D. and repeated in at least three independent experiments. *P*-value < 0.05 was considered statistically significant.

## Results

### p50 was required for HIF-1α protein stabilization upon arsenite exposure

**Fig. 1:**
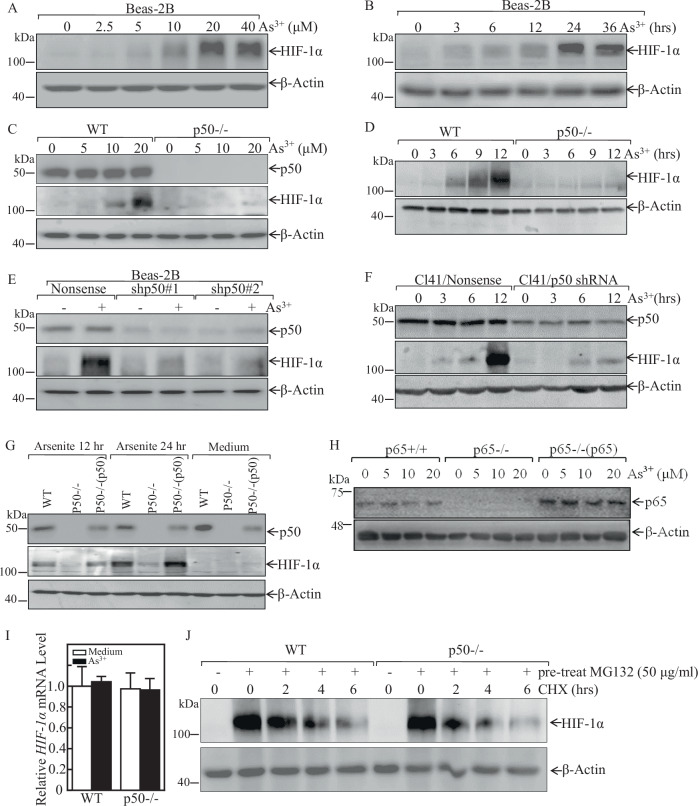
p50, but not p65, was required for arsenite-induced HIF-1α protein expression following arsenite treatment. **A**, **B** Beas-2B cells were treated with various doses of arsenite for 24 h or 20 μM arsenite for the indicated time periods. The cells were then extracted and subjected to western blot assay. **C**, **D** WT and p50^−/−^ cells were exposed to indicated concentration arsenite for 24 h (**C**), or were treated with 20 μM arsenite for the indicated times (**D**). The cells were then extracted and subjected to western blot assay. **E**, **F** Beas-2B cells and mouse epidermal Cl41 cells were stably transfected with shRNA p50, these transfectants were then treated with 20 μM arsenite for 24 h (**E**) or for different time points (**F**) as indicated; The cell extracts were subjected to western blot by using indicated antibodies. **G** WT, p50^−/−^, and p50^−/−^(p50) cells were exposed to 20 μM arsenite for 12 h or 24 h; **H** p65^+/+^, p65^−/−^, and p65^−/−^(p65) cells were treated with arsenite at indicated doses for 24 h. Whole-cell extracts from each of above experiments were subjected to western blot for the determination of protein expression of p50, p65, and HIF-1α. β-Actin was used as protein loading control. **I** WT and p50^−/−^ cells were exposed to 20 μM arsenite and the *HIF-1α* mRNA expression was determined by Real-Time PCR; **J** WT and p50^−/−^ cells were pretreated with 50 μM of MG132 for 10 h, then these cells were exposed to 50 μg/ml CHX for the times indicated after removal of MG132, and the cell extracts were subjected to western blot for the determination of protein expression of HIF-1α. β-Actin was used as protein loading control.

HIF-1α protein surrounding the blood vessels in the tumors of the arsenite-treated mice was found to be increased [28]. Previous studies have revealed that HIF-1α protein expression is elevated upon arsenite exposure in several cells [29]. Similarly, HIF-1α protein level upregulation in dose-dependent and time-dependent manner was observed in human bronchial epithelial cell Beas-2B cells upon arsenic treatment (Fig. 1A, B). To evaluate whether p50 was critical for arsenite-induced HIF-1α protein accumulation following arsenite exposure, HIF-1α expression level was compared between p50^−/−^ cells and wild-type (WT) MEFs cells. As shown in Fig. 1C, D, following arsenite exposure in time- or dose-dependent manner, p50 deletion led to the completely loss of HIF-1α protein induction. Moreover, we transfected p50 short-hairpin RNA (shRNA) plasmids into Beas-2B and normal mouse epidermal JB6 Cl41 cells to test possible implication of p50 in the HIF-1α induction following arsenite exposure. As shown in Fig. 1E, F, p50 knockdown in Beas-2B and Cl41 cells resulted in a markedly reduction of HIF-1α protein expression following arsenite treatment. Further, the critical role of p50 in HIF-1α protein induction was verified by reconstituted expression of p50 in p50^−/−^ cells using Ad-HA-p50, which could restore HIF-1α protein accumulation following arsenite exposure (Fig. 1G). We next determined whether the positive regulatory effect of p50 was NF-κB-transcriptional dependent. To test this, the role of p65 on arsenite-induced HIF-1α protein upregulation was tested. As shown in Fig. 1H, HIF-1α protein expression was comparable among p65^+/+^, p65^−/−^, and p65^−/−^(p65) cells upon arsenite treatment. Therefore, we concluded that arsenite-induced HIF-1α protein accumulation is mainly through a p50-dependent cascade and p65-independent manner.

To investigate the molecular mechanism of p50-mediated HIF-1α upregulation by arsenite, we performed Real-Time PCR to determine whether p50 regulated *HIF-1α* on mRNA level. The results showed that arsenite treatment did not show any observable effect on HIF-1α expression and there also was no observable difference of *HIF-1α* mRNA levels between WT and p50^−/−^ cells following arsenite treatment (Fig. 1I). Therefore, we next evaluated the potential effect of p50 on HIF-1α protein degradation. The results showed that the HIF-1α protein degradative rate was markedly delayed in WT MEFs cells as compared to p50^−/−^ cells (Fig. 1J). These results demonstrated that p50 exerted its effect on upregulating HIF-1α expression via decreasing HIF-1α protein degradation.

### p50 expression exhibited an inhibitory effect on autophagy to stabilize HIF-1α protein following arsenite exposure

**Fig. 2:**
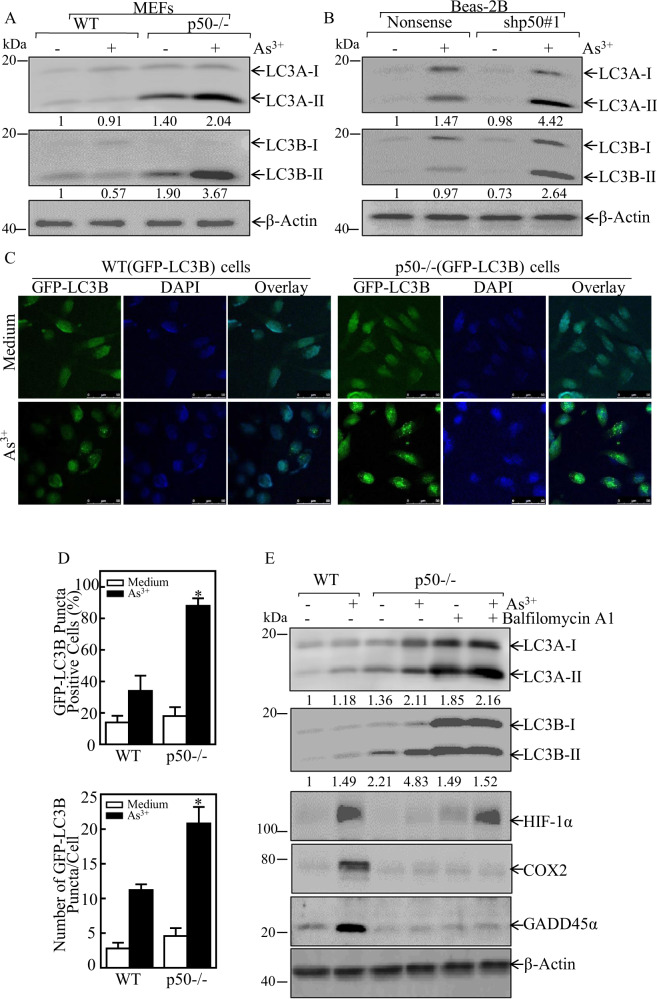
p50 inhibited arsenite-induced autophagy, and in turn resulting in upregulation of HIF-1α protein accumulation following arsenite treatment. **A**, **B** WT, p50^−/−^, Beas-2B(Nonsense), and Beas-2B(shp50) cells were treated with 20 μM arsenite for 24 h. The cells were extracted and cell lysates were subjected to western blot assay by using the indicated antibodies. The ratio of LC3 II/LC3 I was quantified. **C** The GFP-LC3 construct was stably transfected into WT, and p50^−/−^ cells, and the transfectants were treated with 20 μM arsenite for 24 h. LC3 puncta formation was observed and images were captured using fluorescence microscopy. **D** Percentage of cells with GFP-LC3 puncta (in above **C**) and number of puncta per positive cell were calculated and presented. The symbol (*) indicates a significant increase in comparison to WT cells treated with arsenite (*p* < 0.05). **E** WT, and p50^−/−^ cells were treated with 20 μM arsenite, with or without 5 nM BAF for 24 h. the cell extracts were subjected to western blot for the determination of indicated protein expression. The ratio of LC3 II/LC3 I was quantified. β-Actin was used as protein loading control.

Autophagy system senses the external stress signals and induces the lysosomal degradation of futile organelles and proteins [30]. To evaluate the potential mechanism underlying p50 inhibiting HIF-1α protein degradation, we tested autophagy activities in WT and p50^−/−^ cells following arsenite treatment for 24 h. The result showed that p50-deficient led to a remarkably increase in both basal and induced levels of the LC3 conversion from LC3-I to LC3-II in p50^−/−^ cells, as compared to these observed in WT MEF cells (Fig. 2A), suggesting that p50 expression effectively inhibited both basal and arsenite-induced levels of autophagy in the intact cells. This notion was strongly supported by the results that p50 knockdown led to a marked increase of LC3 conversion from LC3-I to LC3-II in Beas-2B cells following arsenite exposure (Fig. 2B).

To further demonstrate autophagy could be induced by arsenite, we stably transfected a GFP-LC3B plasmid into WT and p50^−/−^ cells, which allowed us to observe the formation of autophagosomes. The numbers of GFP-LC3 puncta and the percentage of GFP-LC3 puncta-positive cells were much higher in p50^−/−^(GFP-LC3) cells in comparison to these in WT (GFP-LC3) cells (Fig. 1C, D) following the arsenite treatment for 24 h. The results revealed that the deletion of p50 resulted in the increased of autophagosomes formation and the autophagy induction by arsenite exposure. Most importantly, the reduction of HIF-1α protein accumulation in p50^−/−^ cells was completely rescued by co-treatment of cells with bafilomycin A1 and arsenite. Since our previous studies have demonstrated that p50 is critical for GADD45α induction by arsenite exposure due to its increasing de-ubiquitination of GADD45α protein [10], GADD45α protein was probed as a control in the cells co-treated with BAF. In contrast to HIF-1α, BAF co-treatment showed no observable effect on the defect of GADD45α and COX-2 induction by arsenite in p50^−/−^ cells (Fig. 2E). These results described a specific vital role of autophagy response on the reduction of HIF-1α protein in p50^−/−^ cells with arsenite treatment.

### p50 inhibited both ATG7-mediated and beclin1-mediated autophagy, whereas only ATG7-mediated, but not Beclin1-mediated autophagy was responsible for degradation of HIF-1α protein

**Fig. 3:**
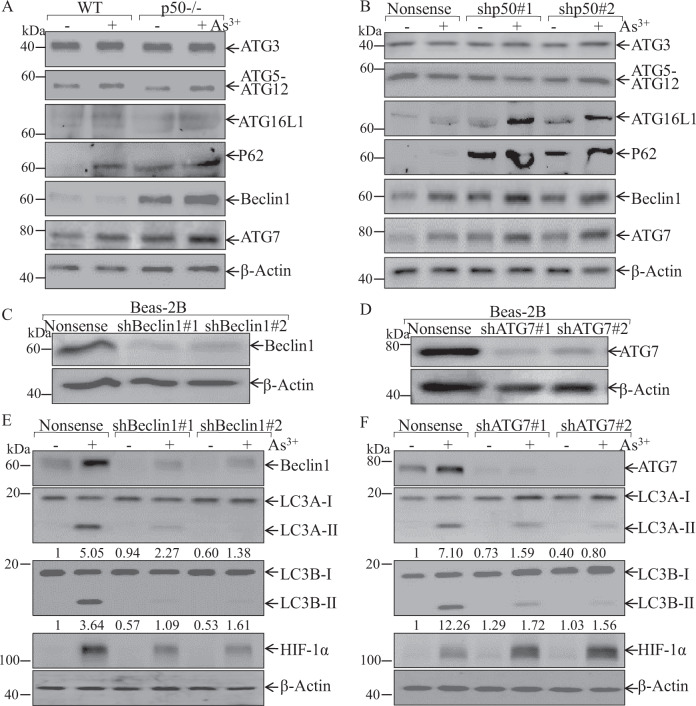
p50 inhibition of autophagy was mediated specifically by attenuation of ATG7, but not Beclin1, following arsenite treatment. **A**, **B** WT, p50^−/−^, Beas-2B(Nonsense), and Beas-2B(shp50) cells were treated with 20 μM arsenite for 24 h as indicated. The cells were extracted and cell lysates were subjected to western blotting assay by using the indicated antibodies. **C**–**F** shRNA ATG7 and shRNA Beclin1 were stably transfected into Beas-2B cells, and the stable transfectants were identified as indicated (**C**, **D**); the stable transfectants were treated with arsenite treatment for 24 h and the cell extracts were subjected to western blot for determination of protein expression by using indicated antibodies, the ratio of LC3 II/LC3 I was quantified (**E**, **F**).

Several central proteins, such as Beclin1, ULK1, and the autophagy-related (ATG) protein family participated in the regulation of cell autophagy in various experimental systems [16]. We next tried to figure out the signaling cascade resulting in the autophagy increased in p50^−/−^ cells due to arsenite exposure. In p50^−/−^ cells, the expression of Beclin1 and the ATG family members, such as ATG3, ATG12-ATG5, ATG16L1, P62, and ATG7 were evaluated in compared with those in WT cells. Both basal and arsenite-induced ATG7, p62, and Beclin1 were markedly increased in p50^−/−^ cells, while ATG3, ATG12-ATG5, and ATG16L1 protein expression did not show observable difference between two types of cells (Fig. 3A). Similarly, knockdown p50 in Beas-2B also promoted ATG7, ATG16L1 and Beclin1 expression as compared to nonsense control transfectants (Fig. 3B), while ATG3, ATG12-ATG5, and P62 protein expression did not show observable difference in both cell transfectants under same experimental conditions, suggesting that ATG7 or Beclin1 might be the players in arsenite-induced autophagy in p50^−/−^ cells.

To evaluate the role of Beclin1 and ATG7 in arsenite-induced autophagy, we transfected short-hairpin RNAs (shRNAs) targeting human Beclin1 (shBeclin1) or ATG7 (shATG7) into Beas-2B cells to knockdown the expression of endogenous Beclin1 or ATG7 (Fig. 3C, D). The result showed that Beclin1 knockdown attenuated the LC3 conversion from LC3-I to LC3-II, but the HIF-1α protein induction was also impaired following arsenite exposure (Fig. 3E). In contrast to Beclin1 knockdown, ATG7 knockdown markedly increased in HIF-1α protein expression in compared with those in nonsense transfectant (Fig. 3F). These data revealed that although p50 inhibited both ATG7-mediated and beclin1-mediated autophagy, only ATG7-mediated, but not Beclin1-mediated, autophagy was responsible for HIF-1α protein degradation following arsenite treatment.

### p50 deletion promoted ATG7 transcription through increasing Sp1 expression

**Fig. 4:**
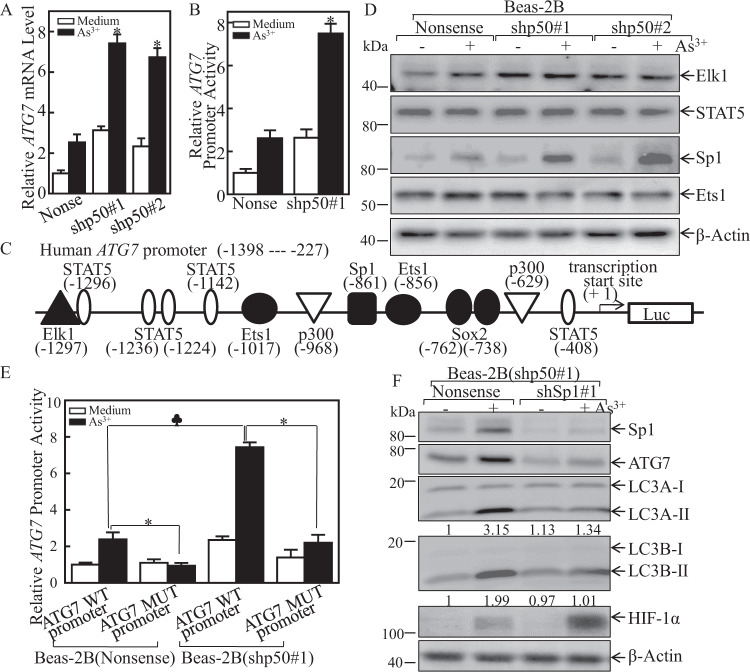
p50 attenuated ATG7 transcription and expression by inhibition of Sp1 expression following arsenite treatment. **A** Beas-2B(Nonsense) and Beas-2B(shp50) cells were treated with 20 μM arsenite and the *ATG7* mRNA expression was determined by Real-Time PCR assay; The symbol (*) indicates a significant increase as comparison to WT cells treated with arsenite (*p* < 0.05). **B** The ATG7 promoter-driven luciferase reporter was transfected into Beas-2B(Nonsense) and Beas-2B(shp50) cells. The stable transfectants were subjected to 20 μM arsenite treatment for 12 h, and the cell extracts were subjected to luciferase activity assay to evaluate relative ATG7 promoter transcriptional activity. The bars show mean ± SD from 3 independent experiments. The symbol (*) indicates a significant increase as comparison to WT cells treated with arsenite (*p* < 0.05). **C** Potential transcription factor binding sites in the ATG7 promoter region (−1398 to −227) were analyzed using the TRANSFAC 8.3 engine online. **D** Beas-2B(Nonsense) and Beas-2B(shp50) cells were treated with 20 μM arsenite for 24 h. The cells were extracted and cell lysates were subjected to western blot by using the indicated antibodies. **E** Wild-type or mutant ATG7 promoter-driven luciferase reporters was co-transfected together with pRL-TK into Beas-2B(Nonsense) and Beas-2B(shp50) cells. 24 h post-transfection, the transfectants were extracted to evaluate the luciferase activity. The results were presented as relative ATG7 promoter activity in response to arsenite exposure. Each bar indicates mean ± SD from 3 independent experiments. The asterisk (*) indicates a significant decrease compared with wild-type reporter transfectant (*p* < 0.05). The spade (♣) indicates a significant increase compared with Beas-2B(Nonsense) cells (*p* < 0.05). **F** The stable transfectants as indicated were exposed to 20 μM arsenite for 24 h. Cell extracts were subjected to Western blot, the ratio of LC3 II/LC3 I was quantified.

To elucidate the molecular mechanism on p50 inhibition of ATG7 expression, we detected *ATG7* mRNA and transcription levels in Beas-2B(nonsense) and Beas-2B(shp50) cells. Consistently, both the *ATG7* mRNA and *ATG7* promoter transcription activity were enhanced in Beas-2B(shp50) as compared to those in Beas-2B(nonsense) cells following arsenite treatment (Fig. 4A, B), conclusively demonstrating that p50 regulating ATG7 expression at transcription level. Using TRANSFAC 8.3 in PROMO HOME PAGE database, we analyzed the potential transcription factor binding sites in ATG7 promoter sequence (Fig. 4C). We further compared those transcription factors expression in Beas-2B(nonsense) and Beas-2B(shp50) cells. The results indicated that p50 knockdown promoted Sp1 expression following arsenite exposure, while it had no effect on the other transcription factors expression, including Elk, Stat5, and Ets (Fig. 4D). These results suggested that Sp1 might be a critical mediator for p50-repressed ATG7 expression. To define the effect of Sp1 on ATG7 transcriptional activity, the Sp1 binding site point mutation in the ATG7 promoter-driven luciferase reporter was constructed. We further transfected both the wild-type or mutant ATG7 promoter-driven luciferase reporters into Beas-2B(Nonsense) and Beas-2B(shp50) cells, and the ATG7 promoter luciferase reporter activity was evaluated. As expected, Sp1 binding site point mutation repressed ATG7 promoter transcription in both transfectants following arsenite exposure (Fig. 4E). Further, shRNA was used to knockdown Sp1 in Beas-2B(shp50) cells, and the Sp1 knockdown efficiency was identified as shown in Fig. 4F. Upon arsenite treatment, Sp1 knockdown in Beas-2B(shp50) cells impaired ATG7 protein expressions, and also attenuated the LC3 conversion from LC3-I to LC3-II, as well as HIF-1α protein accumulation as compared with those observed in the scramble control transfectant, Beas-2B(shp50/nonsense) cells (Fig. 4F). These results clearly exhibited that Sp1 was an p50 downstream effector being responsible for p50 inhibiting ATG7 transcription.

### p50 suppressed *Sp1* mRNA stability through inhibiting Nucleolin (NCL) expression

**Fig. 5:**
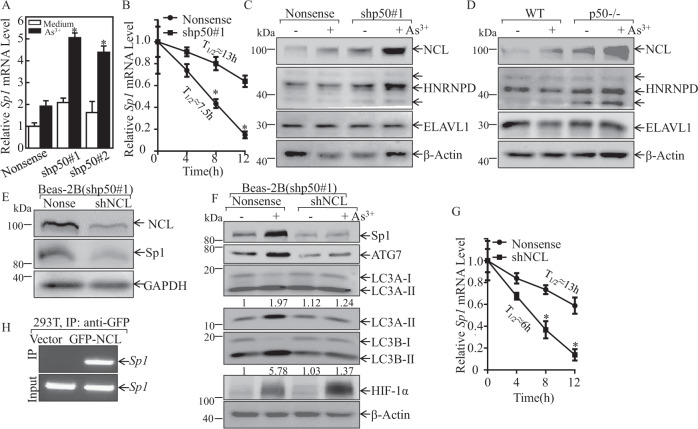
p50 impaired *Sp1* mRNA stability by inhibiting NCL expression upon arsenite treatment. **A** Beas-2B(Nonsense) and Beas-2B(shp50) cells were exposed to 20 μM arsenite and Real-Time PCR assay was performed to evaluate the *Sp1* mRNA expression; The symbol (*) indicates a significant increase as comparison to Beas-2B(Nonsense) cells treated with arsenite (*p* < 0.05). **B** In the presence of actinomycin D (RNA synthesis inhibitor, 10 mM), *Sp1* mRNA degradation rates in transfectants of Beas-2B(Nonsense) and Beas-2B(shp50) were determined for the indicated time periods. **C**, **D** WT, p50^−/−^, Beas-2B(Nonsense), and Beas-2B(shp50) cells were treated with 20 μM arsenite for 24 h. The cell lysates were subjected to western blot. **E**, **F** shRNA NCL was stably transfected into Beas-2B(shp50#1) cells. The indicated stable transfectants were treated with or without arsenite for 24 h; and cell extracts were subjected to Western blot for determination of indicated protein expression, the ratio of LC3 II/LC3 I was quantified. **G** *Sp1* mRNA degradation rates in the indicated cell transfectants were evaluated in the presence of 10 mM actinomycin D (RNA synthesis inhibitor) for the indicated time periods. **H** 293 T cells was transfected with GFP-NCL construct and the cell extracts were subjected to GFP-NCL protein pulled down assay by using anti-GFP beads. RT-PCR was carried out to determine the *Sp1* mRNA bound to NCL protein.

To determine the mechanisms underlying p50 inhibition of Sp1 expression, *Sp1* mRNA were evaluated in Beas-2B(shp50) cell and its nonsense control transfectants following arsenite treatment. Consistently with protein expression, *Sp1* mRNA was upregulated in Beas-2B(shp50) cells in comparison to its nonsense control transfectants following arsenite treatment (Fig. 5A). Further results from analyses of *Sp1* mRNA degradation rates showed that *Sp1* mRNA in Beas-2B(shp50) cells was much more stable than that in Beas-2B(Nonsense) cells upon arsenite exposure (Fig. 5B). These results indicated that p50 played an important role in the suppressing *Sp1* mRNA stability. To determine the upstream mediator for the inhibiting *Sp1* mRNA stabilization by p50, NCL, HNRNPD, and ELAVL1 were tested and compared in Beas-2B(Nonsense) *vs*. Beas-2B(shp50) cells and WT vs. p50^−/−^ cells, following arsenite treatment. While ELAVL1 proteins were comparable, NCL and HNRNPD were elevated in p50 knockdown or knockout cells exposed to arsenite (Fig. 5C, D). These results excluded the role of ELAVL1 and HNRNPD in p50 suppression of *Sp1* mRNA stability. We further knocked down of NCL in Beas-2B(shp50#1) cells and the stable transfectants, Beas-2B(shp50#1/shNCL) and its scramble transfectant Beas-2B(shp50#1/Nonsense), were established and identified (Fig. 5E). Knockdown of NCL dramatically inhibited the induction of Sp1 and ATG7 proteins by arsenite exposure with concurrently attenuating the LC3 conversion from LC3-I to LC3-II, accumulating HIF-1α protein expression (Fig. 5F) and promoting *Sp1* mRNA degradation (Fig. 5G) as compared to the scramble control transfectant, Beas-2B(shp50/nonsense) cells. RNA-IP assay was carried out to evaluate whether NCL was able to directly bind to *Sp1* mRNA, in HEK 293 T cells that expressed GFP-NCL. The results showed that NCL did bind to *Sp1* mRNA (Fig. 5H). Consequently, our results revealed that p50 reduced NCL expression, which leading to impair NCL binding to *Sp1* mRNA, finally resulted in inhibiting *Sp1* mRNA stabilization, and in turn suppressing ATG7 transcription.

### p50 suppressed NCL protein translation through inducing miR-494 expression

**Fig. 6:**
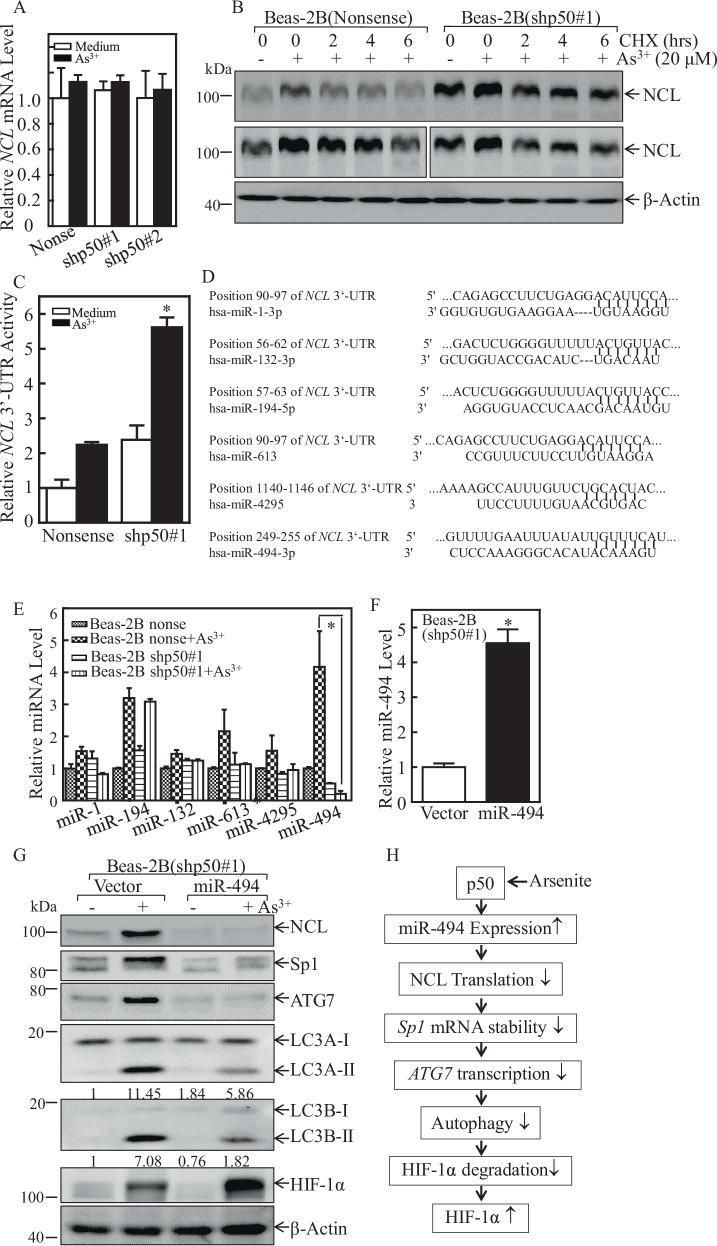
p50-mediated MiR-494 upregulation directly binding to NCL 3’-UTR and attenuated its protein translation and expression upon arsenite treatment. **A** Beas-2B(Nonsense) and Beas-2B(shp50) cells were treated with 20 μM arsenite and Real-Time PCR assay was performed to evaluate the *NCL* mRNA expression. **B** Beas-2B(Nonsense) and Beas-2B(shp50) cells were pretreated with arsenite (20 μM) for 12 h and then exposed to CHX (10 μM) for the indicated time points after the removal of arsenite. The cell extracts were subjected to western blot for determine NCL protein levels. **C** Wild-type *NCL* 3’-UTR-drived luciferase reporters together with pRL-TK were co-transfected into Beas-2B(Nonsense) and Beas-2B(shp50) cells, respectively. The transfectants were treated with arsenite for 24 h, and cell extracts were subjected to the luciferase assay. The results were presented as *NCL* 3’-UTR activity relative to the vector control transfectant, and each bar indicates mean ± SD from 3 independent experiments. **D** The potential miRNA binding sites in the 3’-UTR region of *NCL* mRNA. **E** Real-time PCR was used to determine the expression levels of miRNA in Beas-2B(Nonsense) and Beas-2B(shp50) cells following arsenite exposure. **F** Real-time PCR was performed to evaluate the miR-494 expression in Beas-2B(shp50#1/miR-494) transfectant in comparison to that in Beas-2B(shp50#1/Vector) cells. **G** The indicated stable transfectants were treated with arsenite for 24 h and cell extracts were subjected to western blot for evaluating the effect of miR-494 on expression of NCL, Sp1, ATG7, HIF-1α and the conversion of LC3 from LC3-I to LC3-II. β-Actin was used as a protein loading control, the ratio of LC3 II/LC3 I was quantified. **H** molecular mechanisms underlying p50 promotion of HIF-1α protein accumulation through suppression of ATG7-dependent autophagy.

To elucidate the molecular mechanism of p50 downregulation of NCL expression, the *NCL* mRNA level was evaluated in Beas-2B(shp50) cells and Beas-2B(Nonsense) cells following arsenite exposure. We found that both arsenite treatment and knockdown of p50 showed no effect on *NCL* mRNA expression, as shown in Fig. 6A, excluding the possibility that NCL was regulated at either mRNA stability or transcription. Further, the results from assessing the protein degradation rates indicated that arsenite treatment showed similar effects on inhibition of NCL protein degradation in both Beas-2B(Nonsense) and Beas-2B(shp50) cells (Fig. 6B), revealing that p50 knockdown did not stabilize NCL protein. Thus, we anticipated that p50 might regulate NCL expression at protein translational level. MiRNAs could affect the translation or stability of their targeted mRNAs by directly binding to the 3’-untranslated region (UTR) of the mRNA [31]. Therefore, we next tried to find out the potential differences of *NCL* mRNA 3’-UTR activity in Beas-2B(shp50) cells and Beas-2B(Nonsense) cells following arsenite exposure. The results showed that either basal or arsenite-induced levels of *NCL* mRNA 3’-UTR activity in Beas-2B(shp50#1) cells was much higher than those in Beas-2B(Nonsense) cells (Fig. 6C), indicating that miRNAs might participate in this regulation. Thus, a bioinformatics searching was carried out to find the potential miRNAs that could target *NCL* mRNA 3’-UTR by using TargetScan (v7.0; targetscan.org) [32], PicTar (pictar.org) [33], and miRanda (microrna.org) [34]. The results exhibited that there were numerous putative miRNA binding sites in 3’-UTR region of *NCL* mRNA, such as miR-1, miR-132, miR-194, miR-613, miR-4295, and miR-494 (Fig. 6D). Real-Time PCR was then performed to evaluate the differential expressions of the above miRNAs in Beas-2B(Nonsense) cells and Beas-2B(shp50) cells following arsenite treatment. As shown in Fig. 6E, miR-494 was identified to be upregulated in Beas-2B(Nonsense) cells, whereas it was abolished in Beas-2B(shp50) cells upon arsenite treatment. To identify the effect of miR-494 on the regulation of NCL expression, we transfected a construct expressing miR-494 into Beas-2B(shp50) cells, and the transfectants were identified as shown in Fig. 6F. Upon arsenite treatment, overexpression of miR-494 in Beas-2B(shp50) cells dramatically attenuated the expressions of NCL, Sp1, and ATG7 proteins, decreased the LC3 conversion from LC3-I to LC3-II and increased the accumulation of HIF-1α protein expression (Fig. 6G) as compared to Beas-2B(shp50/Vector) cells. Collectively, our studies discovered that p50 exhibited an inhibition of ATG7-dependent autophagy through miR-494/NCL/Sp1 cascade to directly attenuate HIF-1α protein degradation, thereby leading to HIF-1α protein accumulation following arsenite treatment as schemed in Fig. 6H. Coupled with our previous studies on p50 regulating p53 protein translation and GADD45 protein ubiquitination, our current new findings released the nature of p50 biological effects on the protein expression regulation at multiple levels though NF-κB transcription-independent function.

## Discussion

Arsenite, a human carcinogen, is ubiquitous in the environment, particularly in its contaminated water [35]. Arsenic exposure enhances the transactivation of several transcription factors and their downstream genes to promoting carcinogenesis [36]. HIF-1α can be induced by arsenite, and multiple positive regulators contribute to HIF-1α protein stability and transcriptional activity, such as co-activator proteins and molecular chaperones. It has been reported that arsenic exposure stabilizes HIF-1α protein by promoting reactive oxygen species (ROS) production and inactivate prolyl hydroxylases (PHDs) activity [37]. Our previous studies have discovered that arsenic treatment leads to an increase of HIF-1α protein accumulation through the upregulation of the inducible Hsp70 expression [29]. S6 phosphorylation-dependent manner is responsible for the upregulation of HIF-1α translation, which has been served as a driving force for arsenite-induced cell transformation [38]. Nevertheless, the complex molecular mechanisms underlying arsenite-induced upregulation of HIF-1α protein and activation are still not well understood. Herein, we demonstrated that p50 was much important for arsenite-induced HIF-1α protein accumulation through attenuating ATG7-dependent autophagy mechanism.

Since p50 is a major component of transcription factor NF-κB, p50 exerts its effects predominantly as a heterodimer with RelB, p65, or c-Rel to regulate its downstream gene transcription. Our previous reports have, first time to the best of our knowledge, demonstrated that as lacking the transcriptional domain, a homodimer of p50 exerts its function through transcription-independent mechanisms. We found that by inhibition of GADD45a protein ubiquitination and degradation, p50 is responsible for its upregulating cell apoptosis upon arsenite treatment; [10] p50 modulates the p53 protein expression at the translational level; [14] p50 also impaired c-Myc protein degradation by inhibition FBW7 protein expression [39]. Our present results further showed that p50-mediated HIF-1α protein stabilization/accumulation by the inhibition of ATG7-dependent autophagy, which resulted from the inhibition of NCL/Sp1 expression via upregulation of the miR-494 expression. Arsenite not only is widely used for cancer therapy, but also is a well-recognized human environmental carcinogen, further more mechanisms studies on the function of the p50 will not only shed insight into understanding of the molecular basis for its carcinogenic effect, but may also provide insight into researching of arsenite-p50 downstream mediators and effectors as a potential target for cancer prevention and therapy.

Autophagy has been reported to function as tumor suppressor by delivering damaged organelles/proteins to the lysosome and eliminating genomic instability and inhibiting abnormal cell growth [30, 40]. Alternatively, it also plays a tumor promoting role in several cancers through the intracellular recycling that provides substrates for metabolism and maintains the functional pool of mitochondria [41–43]. Our recent study has discovered that ATG7-mediated autophagy activity is elevated in human bladder cancers, which is crucial for bladder cancer invasion [24] and tumor growth [23]. In this study, inhibition of ATG7-dependent autophagy is found to be responsible for p50-mediated HIF-1α protein stabilization due to arsenite treatment. As a well-known human carcinogen, arsenite has been recognized to regulate the integrity of mammalian cells [44]. Our studies provide significant insight into understanding the nature of the ATG7-mediated autophagic mechanism implicated in arsenite-induced HIF-1α expression, which exhibits a strong possibility for the future development of an autophagy-based-specific therapeutic strategy.

NCL is a multifunctional nucleus protein and is involved in the fundamental regulation of DNA and RNA, such as ribosome biogenesis, mRNA transcription, stability, and translation [45]. In the cytoplasm, nucleolin could interact with mature mRNAs, typically at the 3’-UTR, but sometimes at coding region and the 5’-UTR [46]. Although NCL has critical functions, the mechanisms that control NCL expression are remaining vague. Our studies indicated that p50 exerted inhibition effect on NCL expression, which further attenuated its binding with *Sp1* mRNA, and finally deceased the stability of *Sp1* mRNA following arsenite treatment. RNA-IP assay demonstrated that NCL could directly bind to *Sp1* mRNA and stabilized *Sp1* mRNA, by which mediated ATG7 transcription and expression. Our studies also showed that p50 inhibited NCL protein translation specifically through induction of miR-494 following arsenite treatment.

Our early study has discovered that arsenic promotes human NCL protein SUMOylation at Lys-294 to enhancing *NCL* mRNA binding property by maintaining its nuclear localization [47]. NCL expression has been reported to be positively regulated by ELAVL1, miR-494 could direct bind to NCL 3’-UTR to competition negatively regulate its expression [48]. Consistently, in current study, we identified miR-494 as one of the potent post-transcriptional regulators of NCL. As the data showed in Fig. 6C, although *NCL* mRNA 3’-UTR activity was much higher in Beas-2B(shp50) cells following arsenite exposure, *NCL* mRNA 3’-UTR activity was induced in Beas-2B(nonsense) cells due to arsenite exposure. Moreover, miR-494 expression was induced in Beas-2B(nonsense) cells, while it was inhibited in Beas-2B(shp50) cells with arsenite treatment (Fig. 6E). We supposed that miR-494 is a critical mediator for *NCL* mRNA translation inhibition through binding to *NCL* mRNA 3’-UTR. In addition, further exploring the mechanisms underlying p50 upregulating miR-494 will also be crucial for us fully understanding of p50 nature in regulatory function in the intact cells. Thus, further elucidation of these specific questions are currently ongoing in our research group.

In conclusion, for the first time, our studies discover a novel function of p50 in the HIF-1α stabilization following arsenite treatment through inhibition of ATG7-dependent autophagy. Our studies, coupled with our other previous reports, demonstrate that although p50 lacks its transcription structure, it still programs multiple transcriptional-independent mechanisms to turn on regulation of many key functional proteins expression, by which regulates a few important cellular functions. Moreover, the indicated miR-494/NCL/Sp1/ATG7-dependent autophagy pathway will enrich our understanding of HIF-1α protein degradation regulation, revealing various HIF-1α post-transcriptional regulations under different types of stress.

## Supplementary information

a Reproducibility checklist

Original Data File

## Data Availability

All data generated or analyzed during this study are included in this published article.
