# Correction: NF-κB1 p50 stabilizes HIF-1α protein through suppression of ATG7-dependent autophagy

**DOI:** 10.1038/s41419-024-07279-0

**Published:** 2025-01-27

**Authors:** Junlan Zhu, Shirui Huang, Yang Li, Jiheng Xu, Ruifan Chen, Mengxin Guo, Xiaohui Qian, Tengda Li, Zhongxian Tian, Honglei Jin, Chuanshu Huang

**Affiliations:** 1 Oujiang Laboratory (Zhejiang Lab for Regenerative Medicine, Vision and Brain Health), Key Laboratory of Laboratory Medicine, Ministry of Education, School of Laboratory Medicine and Life Sciences, Wenzhou Medical University, 325035 Wenzhou, Zhejiang China; 2 Precision Medicine Laboratory, Beilun People’s Hospital, Beilun Branch of the First Affiliated Hospital, School of Medicine, Zhejiang University, 315800 Ningbo, Zhejiang China

**Keywords:** Macroautophagy, Transcriptional regulatory elements

Correction to: *Cell Death & Disease* (2022) 13:1076; 10.1038/s41419-022-05521-1; published online 27 December 2022

In the originally published article, there are two errors found in Figure 1H panels and the text in “MATERIALS AND METHODS”: the western blot results of p65 protein expression, the protein loading control β-Actin, and the primer used in this study have been wrongly placed. The correct figure panels and text are presented below. The primers used in this study were: mouse *hif-1α* (Forward, 5′-AGC CCT AGA TGG CTT TGT GA-3′; Reverse, 5′-TAT CGA GGC TGT GTC GAC TG-3′), mouse HIF-1α (Forward, 5′-AGC CCT AGA TGG CTT TGT GA-3′; Reverse, 5′-TAT CGA GGC TGT GTC GAC TG-3′), mouse β-Actin (Forward, 5′-CAT CCG TAA AGA CTC CTA TGC C-3′; Reverse, 5′-ACG CAG CTC AGT AAC AGT CC-3′), human ATG7 (Forward, 5′-GCC AAG ATC TCC TAC TCC AATC-3′; Reverse, 5′-CAG AAG TAG CAG CCA AGC TTGT-3′), human Sp1 (Forward, 5′-GCT ATG CCA AAC CTA CTC CA-3′; Reverse，5′-TGA TCG TGA CTG CCT GAG A-3′), human NCL (Forward, forward: 5′-ACC TAA TGC CAG AAG CCA GCC A-3′; Reverse: 5′-TTG CCC GAA CGG AGC CGT C-3′), and human β-Actin (Forward, 5’-CTC CAT CCT GGC CTC GCT GT-3’; Reverse, 5’-GCT GTC ACC TTC ACC GTT CC-3’). The plasmid of NCL 3’-UTR luciferase reporter was constructed using primers: Forward, 5’-CCG CTC GAG TCC CTC TGC TTT CCC TTT-3’ and Reverse, 5’-CCC AAG CTT GAC TTG GCT ACC TAC ATT GA-3’ and then subcloned into the Xho I and Hind III sites into pMIR vector.
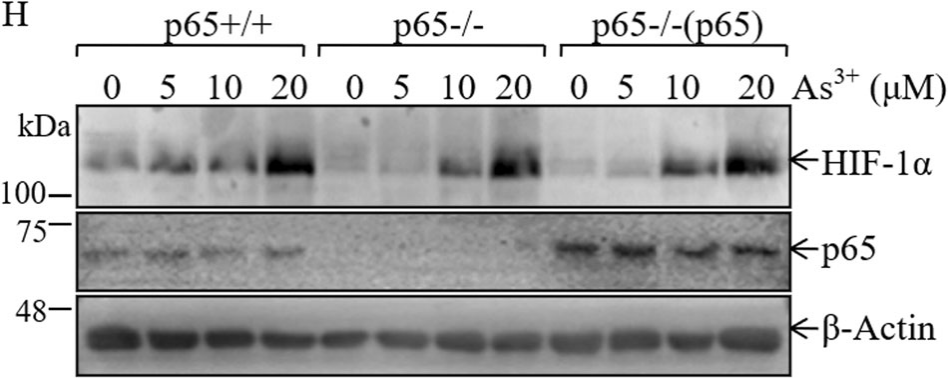

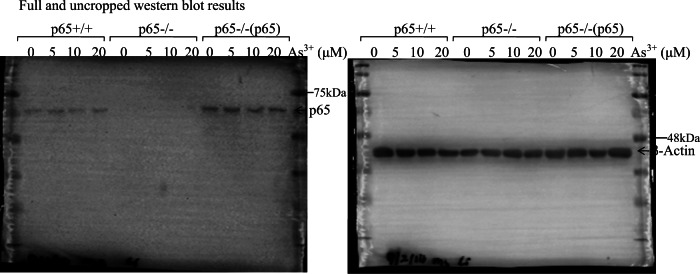


The corrections do not affect the results or conclusions of this work. The authors apologize for any inconvenience this may have caused.

The original article has been corrected.

