# Supplementary figures and images for: NF-κB1 p50 stabilizes HIF-1α protein through suppression of ATG7-dependent autophagy
Source: Cell Death Dis. 2022 Dec 27;13(12):1076. doi: 10.1038/s41419-022-05521-1 (PMC9794792; doi:10.1038/s41419-022-05521-1)

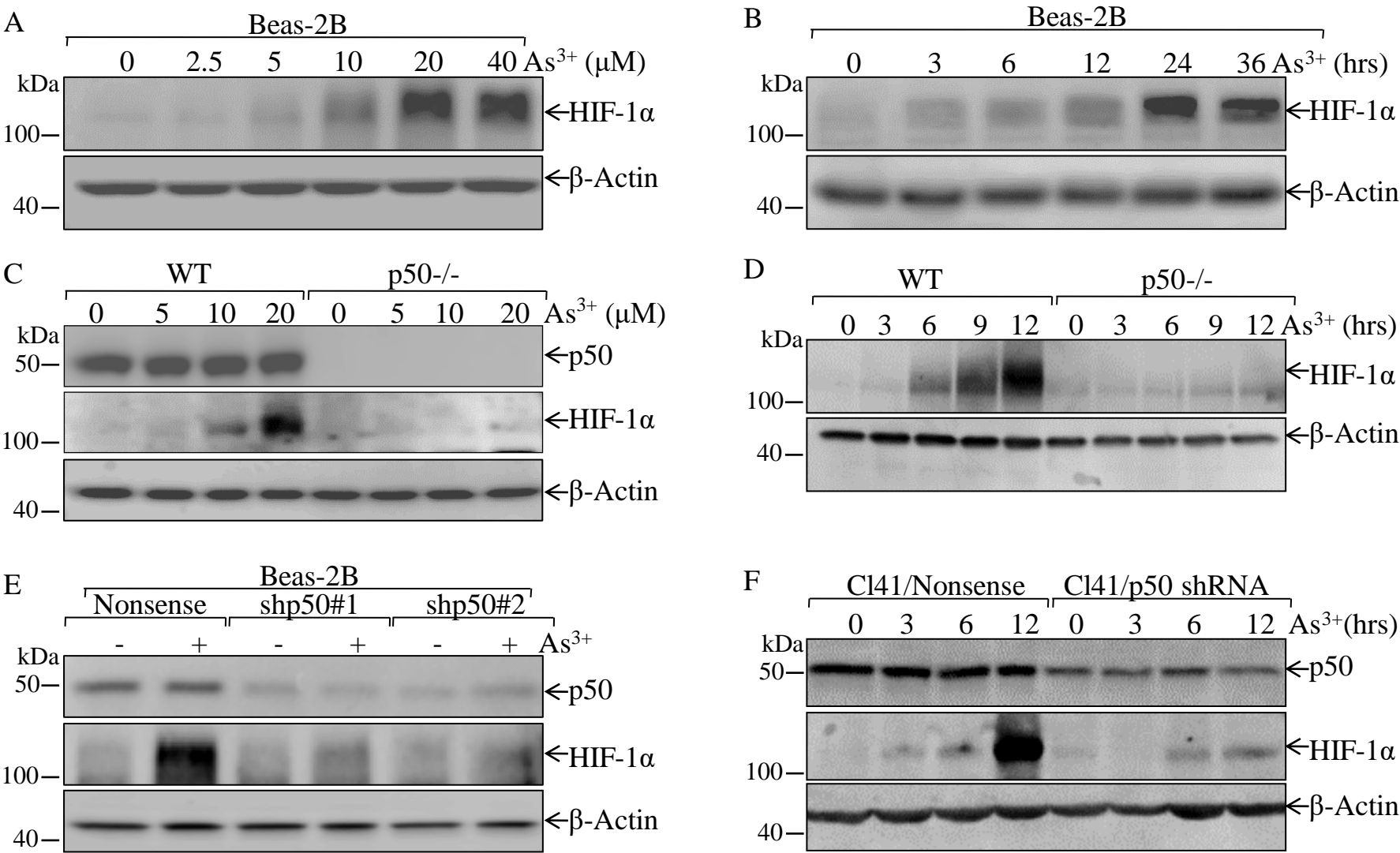

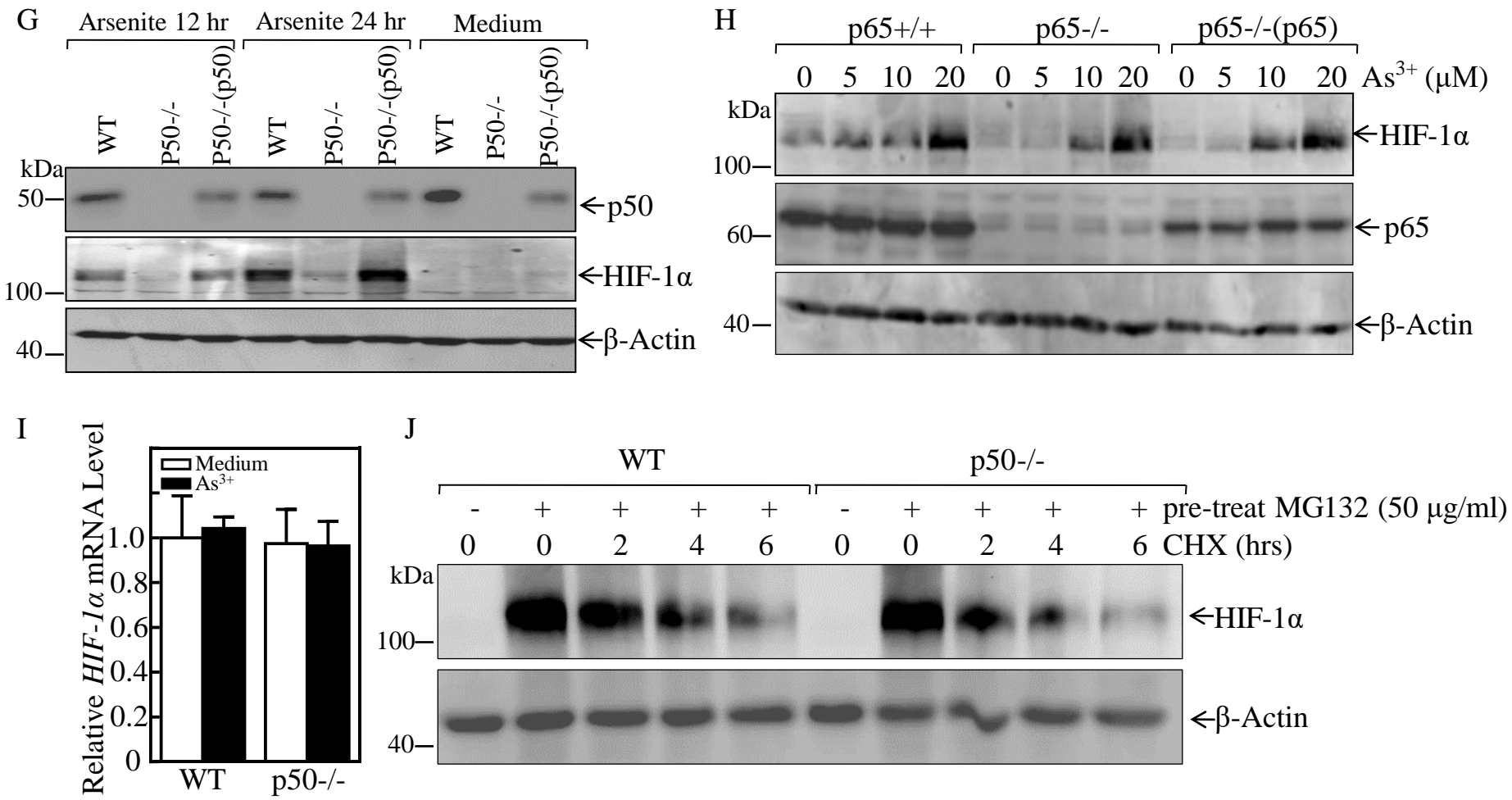

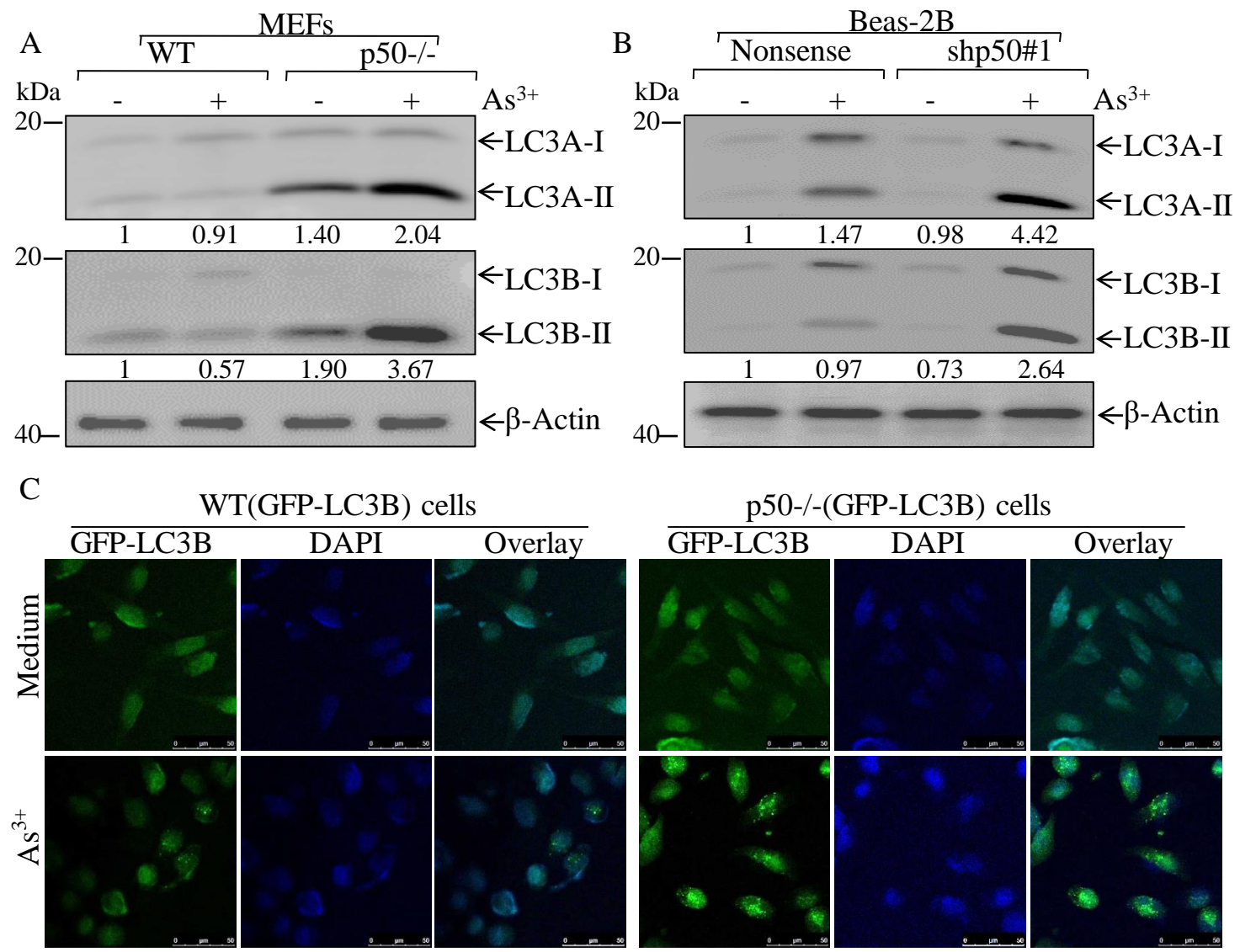

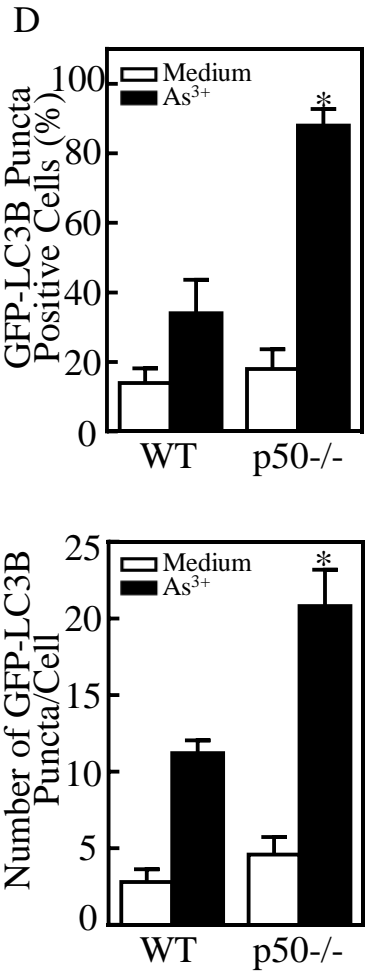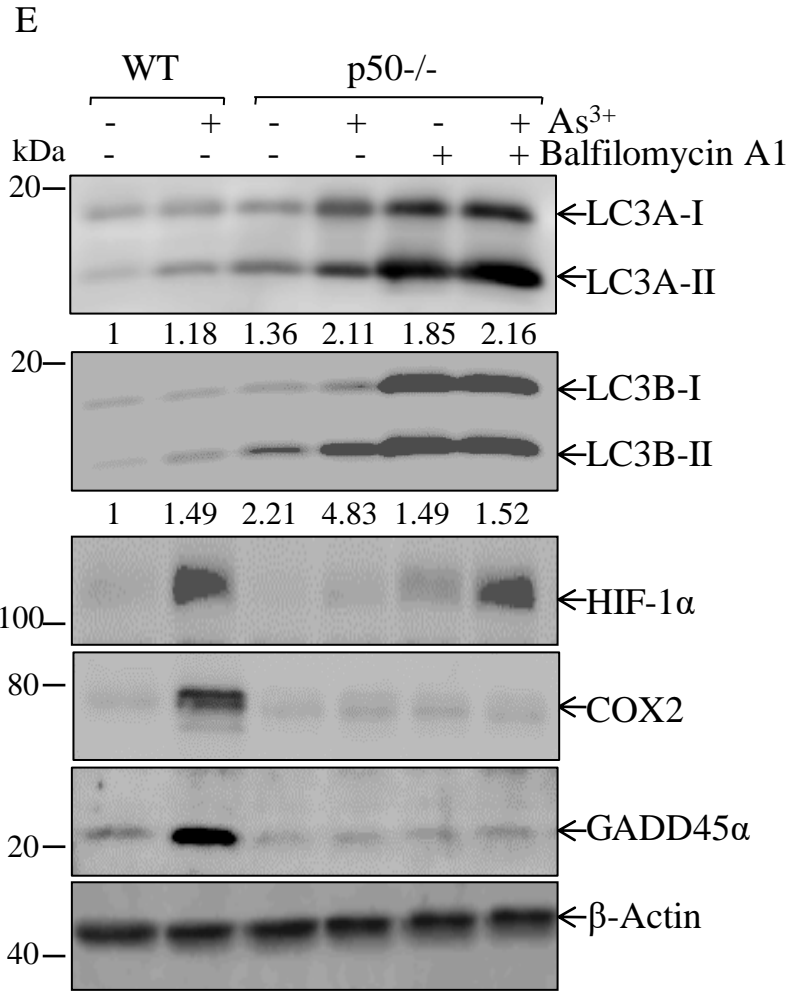

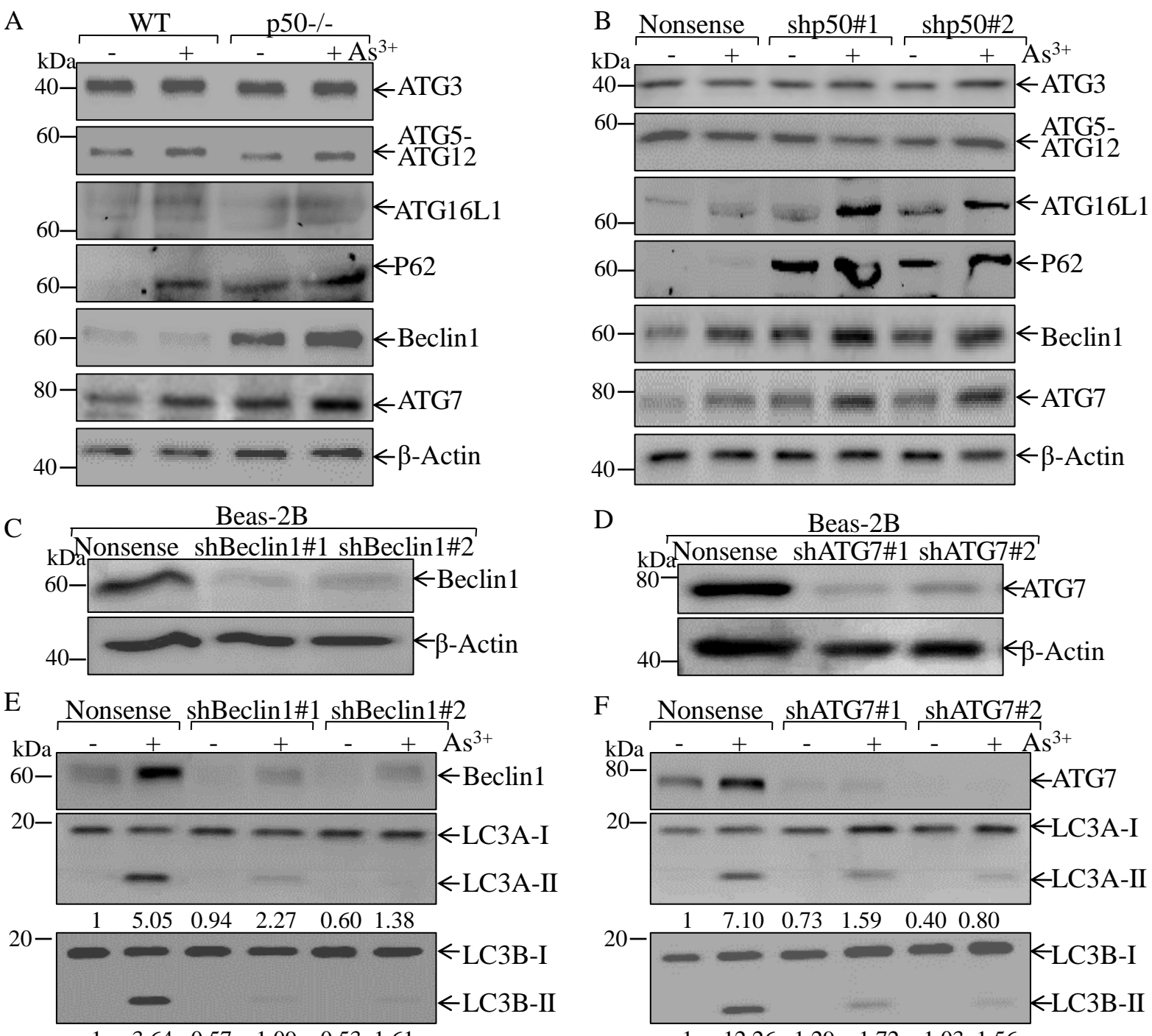

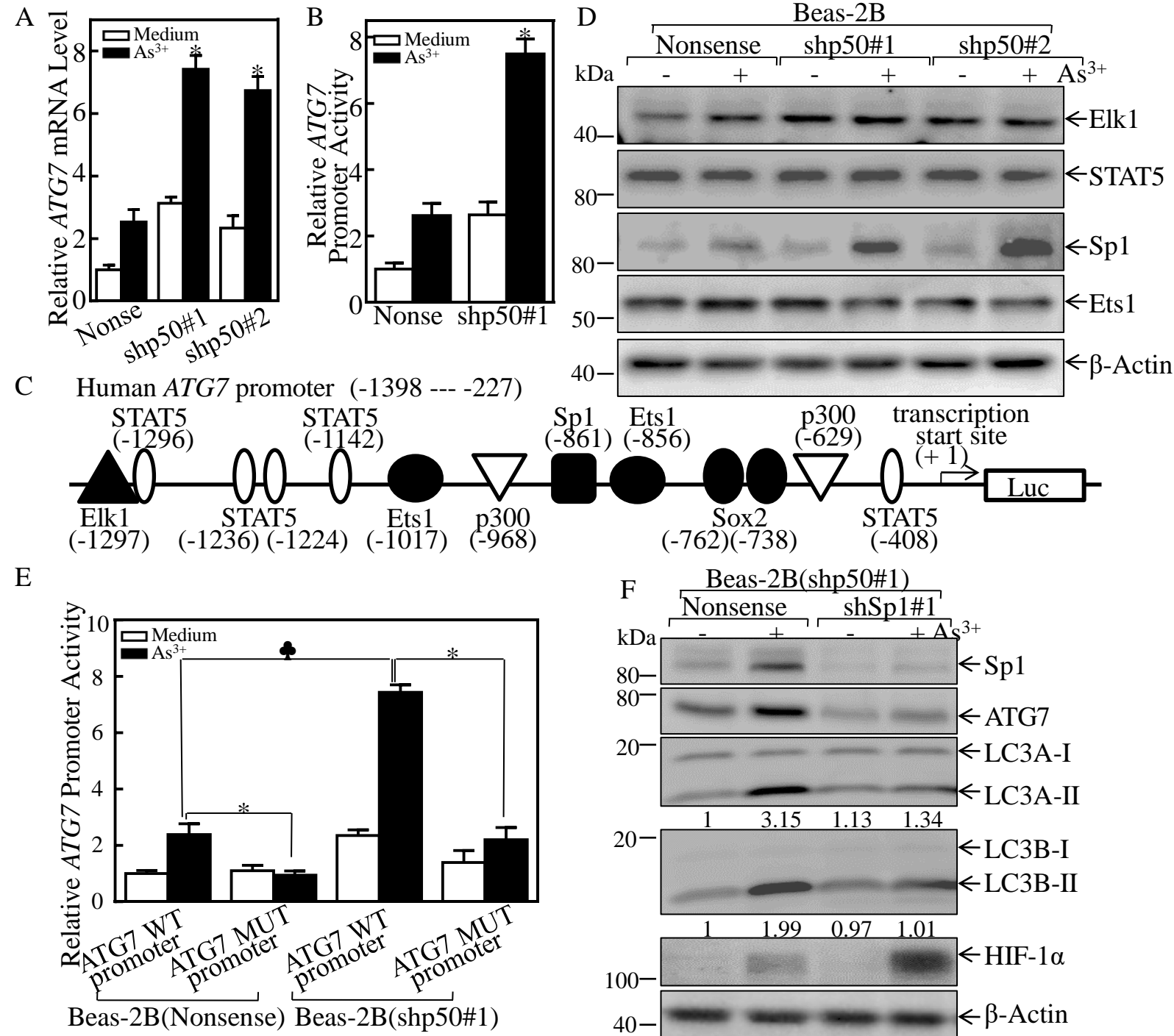

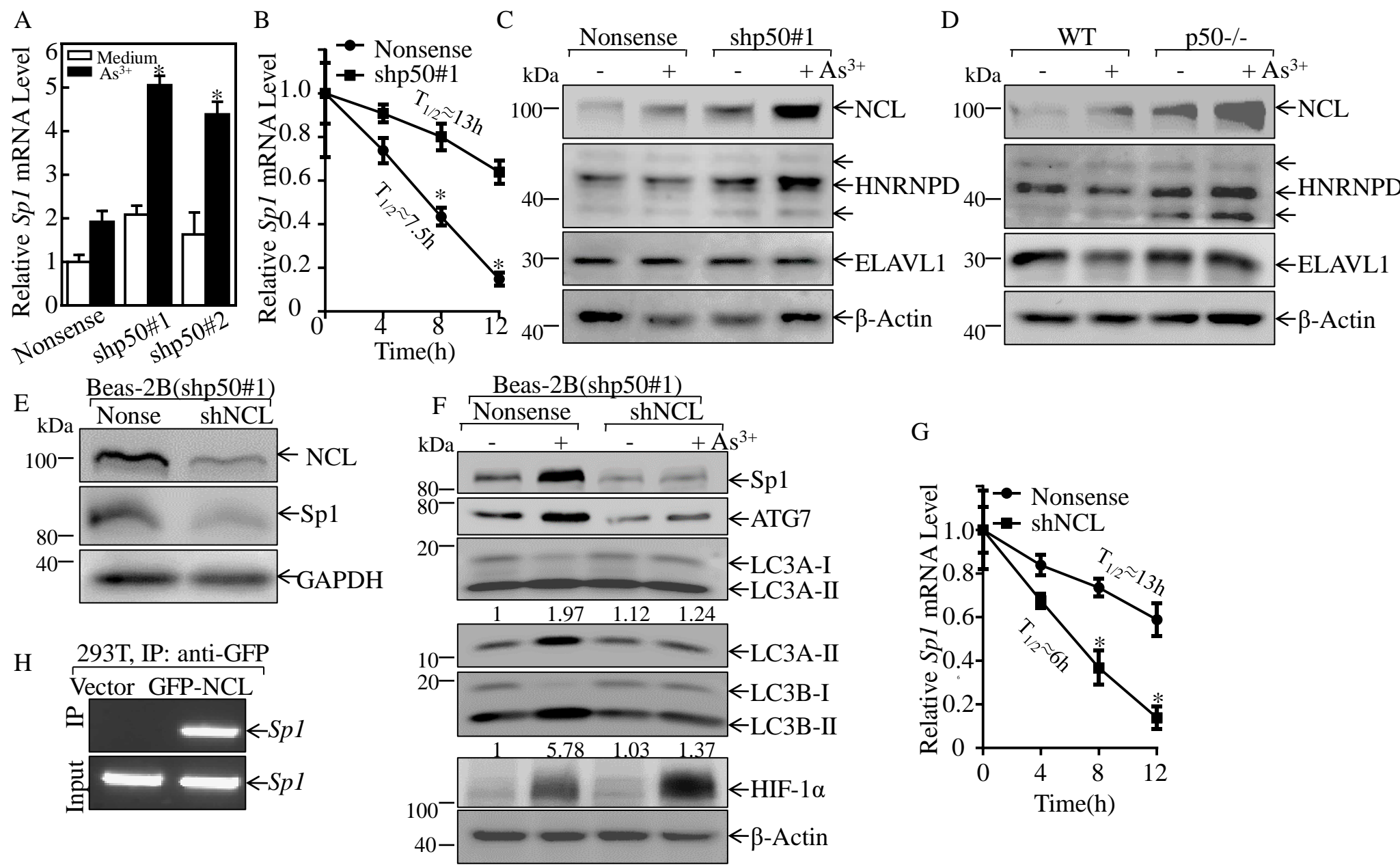

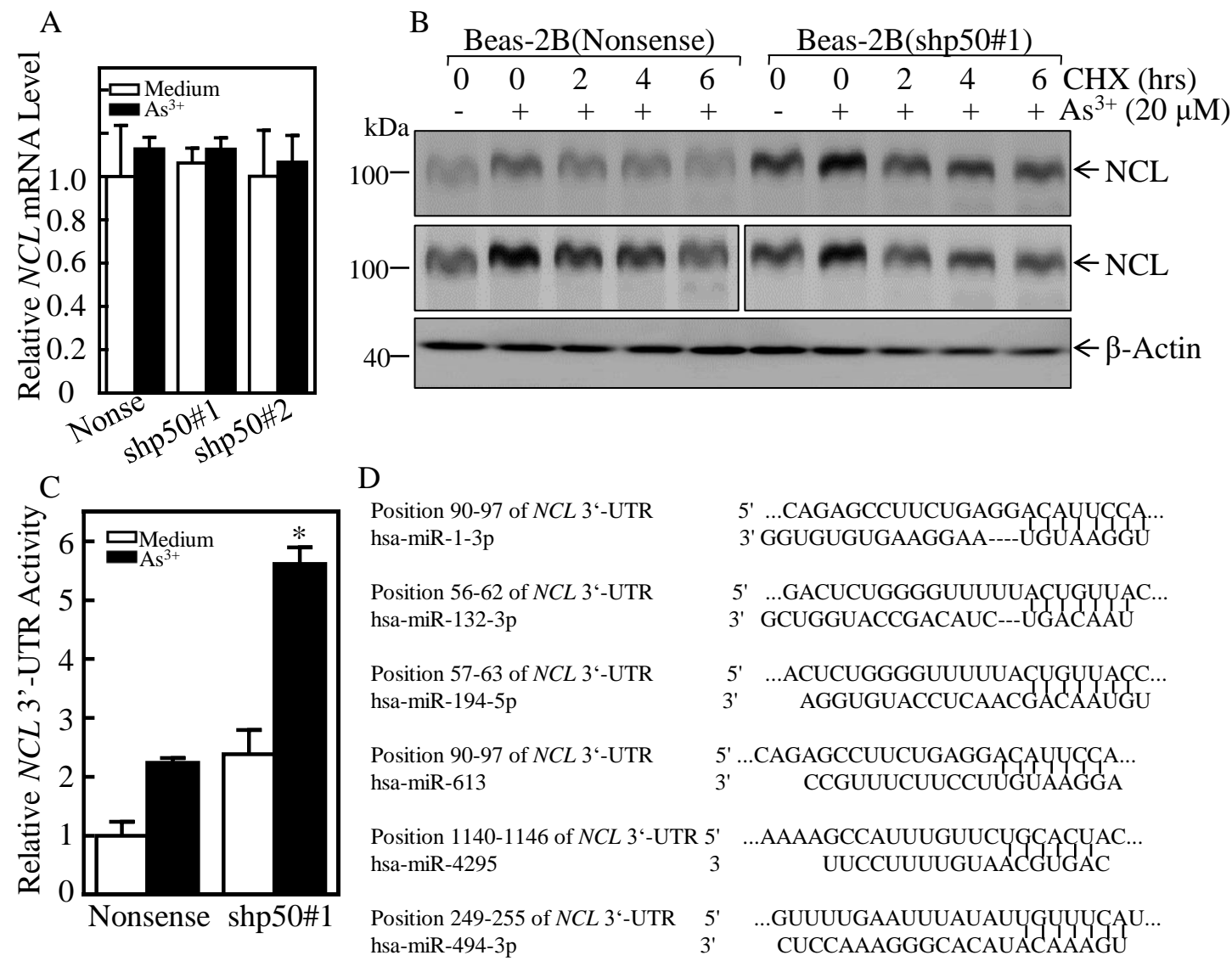

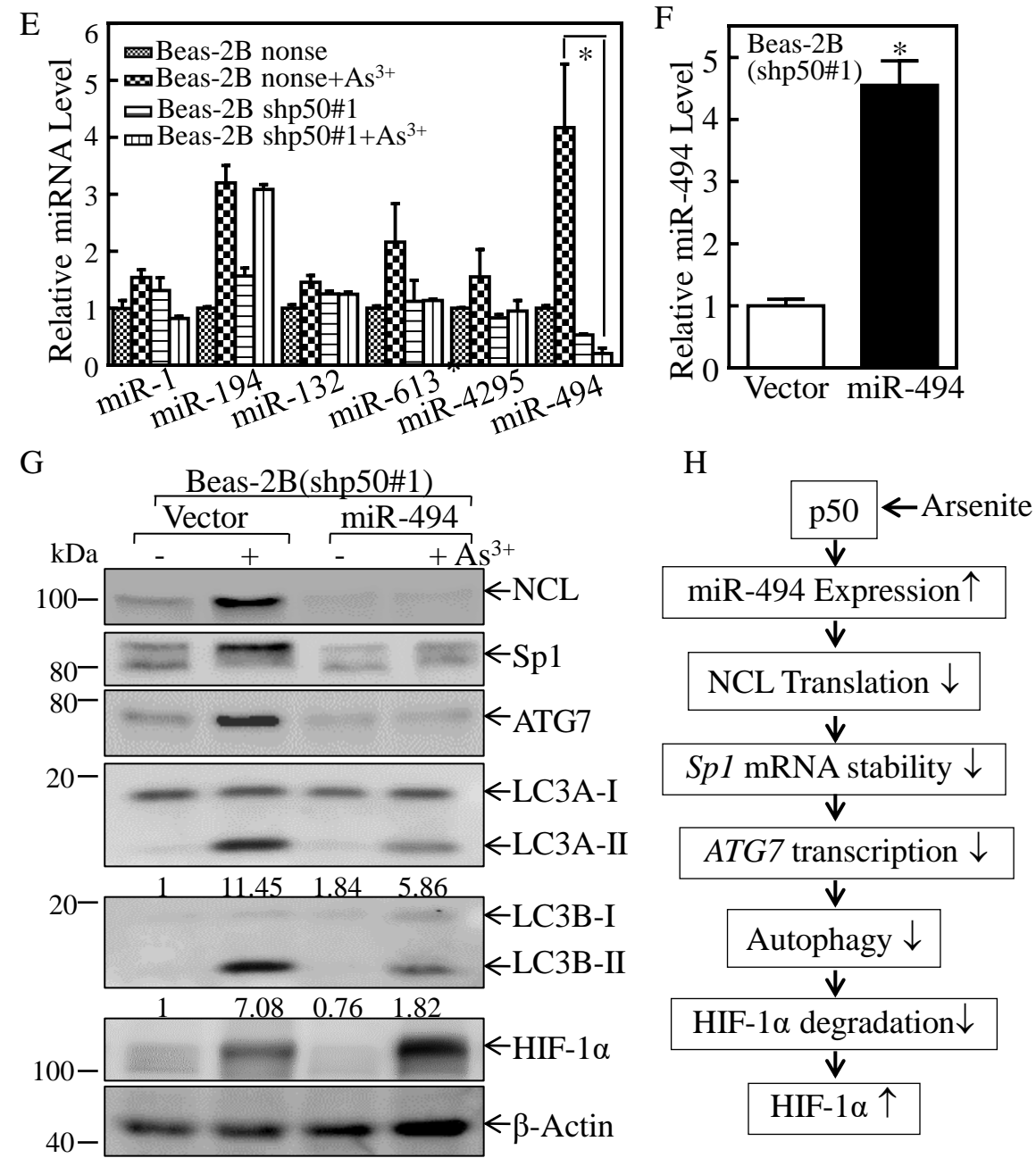

Supplement: Supplementary file 2 — Original Data File [file 41419_2022_5521_MOESM2_ESM.pdf]
